# Evolution of Biosensors and Current State-of-the-Art Applications in Diabetes Control

**DOI:** 10.3390/bios16010039

**Published:** 2026-01-03

**Authors:** Yahya Waly, Abdullah Hussain, Abdulrahman Al-Majmuei, Mohammad Alatoom, Ahmed J. Alaraibi, Ahmed Alaysereen, G. Roshan Deen

**Affiliations:** Materials for Medicine Research Group, School of Medicine, Royal College of Surgeons in Ireland, Medical University of Bahrain, Busaiteen 228, Bahrain; 22200270@rcsi.com (Y.W.); 22200254@rcsi.com (A.H.); 22200036@rcsi.com (A.A.-M.); 22202008@rcsi.com (M.A.); 22202111@rcsi.com (A.J.A.); 22202180@rcsi.com (A.A.)

**Keywords:** biosensors, glucose monitoring, nanotechnology, non-invasive, detection, diabetes management

## Abstract

Diabetes is a chronic metabolic disorder that poses a growing global health challenge, currently affecting nearly 500 million people. Over the past four decades, the rising prevalence of diabetes has highlighted the urgent need for innovations in monitoring and management. Traditional enzymatic methods, including those using glucose oxidase, glucose dehydrogenase, and hexokinase, are widely adopted due to their specificity and relative ease of use. However, they are hindered by issues of instability, environmental sensitivity, and interference from other biomolecules. Non-enzymatic sensors, which employ metals and nanomaterials for the direct oxidation of glucose, offer an attractive alternative. These platforms demonstrate higher sensitivity and cost-effectiveness, though they remain under refinement for routine use. Non-invasive glucose detection represents a futuristic leap in diabetes care. By leveraging alternative biofluids such as saliva, tears, sweat, and breath, these methods promise enhanced patient comfort and compliance. Nonetheless, their limited sensitivity continues to challenge widespread adoption. Looking forward, the integration of nanotechnology, wearable biosensors, and artificial intelligence paves the way for personalized, affordable, and patient-centered diabetes management, marking a transformative era in healthcare. This review explores the evolution of glucose monitoring, from early chemical assays to advanced state-of-the-art nanotechnology-based approaches.

## 1. Introduction

Diabetes has had a significant increase in prevalence within the past 40 years, according to the International Diabetes Federation (IDF). Within the year 2021, there were an estimated 537 million people worldwide who were living with diabetes [1]. Moreover, the World Health Organization (WHO) now considers diabetes to be an epidemic and one of the leading causes of mortality worldwide [2].

Several studies, including those conducted by the Mayo Clinic, have illustrated that diabetes is associated with a range of serious complications, such as cardiovascular disease, diabetic neuropathy, diabetic retinopathy, and Alzheimer’s disease [3]. Over the past decades, various detection methods have been developed to ensure early diagnosis, as early diagnosis is associated with an improved prognosis. These methods include biochemical tests (e.g., FPG test, OGTT, HbA1c), continuous glucose monitoring (CGM), and non-invasive technologies (e.g., transdermal patches and breath analyzers). Among these detection methods, biosensors are particularly beneficial for detecting and managing diabetes as they can improve glycemic control through continuous real-time monitoring [4].

Given the rising prevalence and significant burden of diabetes, it is important to examine the methods of utilizing biosensors for its detection and management. This article aims to achieve this by analyzing key components, including the historical development of glucose detection, the different types of biosensor detection methods, and the future directions in glucose monitoring technology.

## 2. Historical Development of Glucose Detection Methods

To understand the advancements and current state of glucose detection, it is important to consider its historical development from the rudimentary methods of the early 19th century to the more sophisticated technologies found in present-day health sciences. The goal is to understand the evolutionary timeline of diabetes management, while pinpointing valuable lessons for ongoing development in the field.

### 2.1. Early Methods and Chemical Tests

The foundation for glucose detection was largely built on the works of Hermann Von Fehling and Stanley Benedict, both of whom were essential in the initial efforts to measure glucose levels in the human body. In 1841, Fehling introduced Fehling’s solution, a chemical reagent used to distinguish between water-soluble carbohydrates and ketone functional groups [5]. When heated with a glucose-containing sample, Fehling’s solution produced a red precipitate of copper (I) oxide, indicating the presence of reducing sugars such as glucose [6]. Building on Fehling’s work, Stanley Benedict developed the Benedict’s Test in 1908 [7]. This test offered a more streamlined approach to detecting glucose in urine by relying on the reduction of copper (II) sulfate to copper (I) oxide in the presence of reducing sugars. While these chemical tests were transformative to the way scientists approach glucose monitoring, they were largely qualitative and lacked precision, prompting further development in the field.

### 2.2. Advancement to Portable Devices

The 1970s marked a significant leap with the development of the first portable blood glucose meter, the Ames Reflectance Meter (ARM), introduced by Anton H. Clemens [8]. This device welcomed an era of at-home glucose monitoring that provided diabetic patients with greater autonomy and control over their condition. To achieve this, ARM devices utilized Dextrostix, a paper strip coated with glucose oxidase, peroxidase, and chromogen that reacted with a sample of the user’s blood, producing a color change that would indicate their blood glucose levels [8].

A decade later, these methods were further refined with the advent of user-friendly glucose monitors, including the Accu-Chek by Roche and the OneTouch by Lifescan, both of which were found to be more accurate and convenient compared to their predecessors [6]. Their operational design involved the use of electrochemical detection methods whereby glucose in the blood would react with an enzyme electrode, generating an electrical current proportional to the user’s glucose concentration [9].

### 2.3. Continuous Glucose Monitoring

In the late 1990s and early 2000s, CGM systems were introduced [10]. These systems significantly improved diabetes management by using a small sensor under the skin to measure interstitial glucose levels continuously, providing real-time readings, trends and alerts [10]. CGMs were further refined in the 2010s, bringing us more advanced systems such as the Dexcom G6 and FreeStyle Libre. While these models also involve inserting a small sensor under the skin, they do not require routine fingerstick calibrations, making them nearly prick-free [5]. In 2024, CGM systems remained the predominant devices for glucose monitoring globally due to their convenience and minimal invasiveness [11]. However, with patient comfort and compliance at the forefront, research and development into non-invasive glucose monitoring technologies appear to be the focus moving forward, with optical, microwave, and electrochemical sensors being a point of emphasis in recent studies [12,13,14].

In the late 1990s and early 2000s, continuous glucose monitoring (CGM) systems were introduced, representing a major advance in diabetes management [10]. These systems use a small sensor placed in the subcutaneous tissue to measure interstitial glucose levels continuously, providing real-time readings, trend analysis, and alerts for hypo- and hyperglycaemia [10]. This continuous data allows for improved glycaemic control compared with intermittent finger-prick testing [10].

Over time, CGM technology evolved toward more user-friendly and less invasive designs. Widely adopted systems such as the Dexcom G6 and FreeStyle Libre utilise minimally invasive subcutaneous sensors and eliminate the need for routine finger-stick calibration, significantly improving patient comfort and adherence [5]. These wearable CGM platforms currently dominate clinical practice due to their reliability, convenience, and integration with smartphone-based health ecosystems [11].

More recently, alternative CGM designs have emerged that represent distinct future directions in glucose sensing. Implantable CGM systems, such as the Senseonics Eversense, involve a fully implanted sensor placed subcutaneously for long-term glucose monitoring over several months [12,13,14]. These systems offer extended sensor longevity and reduced user maintenance, highlighting a shift toward implant-based glucose sensing technologies. In contrast, newer wearable approaches continue to focus on minimising invasiveness. In 2024, the BiolinQ system received regulatory approval as the first microneedle-based dermal glucose sensor, using an array of microscopic needles that penetrate the superficial layers of the skin without reaching nerve-rich tissue [15]. This approach represents a hybrid between traditional subcutaneous sensors and non-invasive monitoring, offering a promising balance between accuracy and patient comfort.

Together, these developments illustrate the diversification of CGM technologies into implantable and advanced wearable platforms. While CGM systems remain the predominant method of glucose monitoring globally, ongoing innovation suggests a future pipeline that includes long-term implantable sensors, microneedle-based dermal devices, and ultimately fully non-invasive glucose monitoring technologies [16].

## 3. Parameters That Define Effective Glucose Detection

The effectiveness of glucose monitoring systems is primarily defined by their accuracy and precision, which ensure consistent and reliable measurements [17,18]. Other key parameters include sensitivity and specificity, essential for detecting low glucose levels such as in hypoglycemia and for avoiding interference from other blood components [19]. Timeliness of detection and ease of use, especially in portable and continuous monitoring devices, further enhance clinical decision-making and home-based diabetes management [18]. Advances from early chemical tests to modern continuous monitoring systems have significantly improved the accuracy, convenience, and integration of glucose monitoring into digital health ecosystems [18].

The effectiveness of glucose monitoring systems can be evaluated using both academic performance metrics and clinically regulated industry standards [17,18]. In this review, the parameters discussed are framed primarily from a clinical and translational perspective, reflecting the requirements used to validate glucose-sensing technologies intended for patient use [19]. Regulatory bodies and international standards organisations define specific performance benchmarks that must be met before glucose monitoring devices can be approved for clinical practice, including accuracy, precision, sensitivity, specificity, response time, and long-term reliability [18].

Accuracy is most commonly assessed using the Mean Absolute Relative Difference (MARD), which quantifies the average deviation between sensor measurements and reference glucose values, and is widely accepted as a primary indicator of CGM performance in regulatory and clinical evaluations [20]. In addition, error grid analyses, such as the Clarke Error Grid and the Continuous Glucose Error Grid Analysis, are used to evaluate the clinical significance of measurement errors by categorising glucose readings according to their potential impact on patient decision-making [21,22]. These tools are central to regulatory assessment, as they move beyond analytical accuracy to determine whether sensor errors could lead to inappropriate clinical actions.

Other key parameters include sensitivity and specificity, which are critical for detecting clinically significant glucose excursions such as hypoglycaemia, as well as timeliness of detection and ease of use, which directly influence patient adherence and real-world effectiveness [8,23]. Modern regulatory frameworks, including the International Organization for Standardization standard ISO 15197, emphasise that glucose monitoring technologies must demonstrate both analytical accuracy and clinical safety to support reliable patient care [24]. By aligning performance metrics with these regulatory standards, glucose sensing technologies can more effectively transition from laboratory development to routine clinical application.

## 4. Nanotechnology

Today, biosensors are used in a wide range of industries, including food processing, biomedicine, healthcare, and tissue engineering. They are used in diagnostic procedures as well as for the detection of various biological components. Biosensors are a promising diagnostic tool due to their unique electrochemical properties [25]. According to the International Union of Pure and Applied Chemistry (IUPAC), a biosensor is a device that uses a precise biochemical reaction, arbitrated by the immune system, isolated enzymes, organelles, or tissues for the detection of chemical compounds [26]. This section will concentrate on nanoparticles, their functions, and how they affect the other types. The synthesis of nano-biosensors commonly makes use of nanomaterials, including nanoparticles, nanowires, carbon nanotubes (CNTs), nanorods, and QDs, which acquire information at atomic and subatomic scales, allowing them to detect microscopic items [25,27].

### 4.1. Types of Nano-Biosensors

A typical biosensor’s primary structure consists of the following five parts: (A) Analyte—the molecule or chemical that is measured or found. (B) Bioreceptors—Detects analyte interaction by generating a change in light, heat, pH, or sound after coming in contact with the analyte. (C) Transducer—transforms energy into a quantifiable form by changing its shape. (D) Electronics—these take the signal from the transducer, process it, amplify it, and turn it into electricity. (E) a display that shows the result and the device, such as a computer or printer [28].

The following factors can be used to categorize nanobiosensors: the bioreceptor used, the energy source (which can include a piezoelectric thermocouple and sensor), the application of the nanobiosensor (which can consist of chemical, biosensor, deployable, and electrometer sensors), and the basis of the structure that is related to the transducer type [27].

### 4.2. Carbon-Based Nanobiosensors

The broad range of carbon nanomaterials, including CDs, CNTs, graphene/graphene oxide, and graphene quantum dots, has been utilized to construct high-performance glucose fluorescent nanobiosensor assays and devices. Moreover, nanotechnology has been integrated in fluorescent biosensors, including semiconductor quantum dots, mesoporous silica nanomaterials, upconverting nanomaterials, plasmonic gold/silver nanoparticles, and fluorescent gold/silver quantum clusters [29]. An example of the application of nanotechnology in fluorescent biosensors is the usage of QDs. Because of quantum confinement effects, QDs with a diameter of 2–10 nm [29] can have special optical characteristics. It offers size-tunable photoluminescence, a broad excitation range accompanied by a narrow emission peak, a significant Stokes shift, ultrahigh brightness, and high photostability [29]. This property offers multiple advantages to glucose-sensing systems, as it enables the simultaneous detection of various analytes, a process known as multiplexing [30,31]. Furthermore, it provides size-tunable bright emission, broad excitation flexibility, high photostability, and reliable long-term, excellent signal-to-noise performance, which can boost the sensitivity and specificity of the sensors [31].

### 4.3. Quantum Dots (QDs)

The QD system can detect glucose in the body through the Förster resonance energy transfer (FRET) mechanisms. Tang et al. developed a nano biosensor using cadmium telluride quantum dots (CdTe QDs) and gold nanoparticles (AuNPs) [32]. An assembled QDs-ConA-β-CDs-AuNPs system, where concanavalin A (ConA) binds to β-cyclodextrins (β-CDs), brings the QD donor and AuNP acceptor into proximity, quenching the QD fluorescence. The glucose competes with β-CDs, causing it to displace and restore fluorescence, thereby enabling the detection of glucose with a detection limit of 50 nM and high sensitivity. It demonstrated excellent selectivity over interfering species and approximately 90% fluorescence recovery, indicating its potential use in biological applications [32]. QDs have several benefits over organic fluorophores. QDs exhibit long fluorescence lifetimes, significant Stokes shifts, high molar extinction coefficients, broad excitation spectra, and tunable emission spectra which lead to improved photostability, background autofluorescence removal, and multicolor imaging [33]. QDs, however, do have several drawbacks, such as intermittent luminescence and possible toxicity, which may be overcome by additional engineering and different production techniques, such as adding a coat of carbon, silica, or polymer to make it more biocompatible and water-soluble [29].

### 4.4. Silver-Based Nanoparticles in Nanobiosensor

Silver-functionalized bismuth oxide (AgBi_2_O_3_) nanoparticles (SBO NPs) were found to be effective in monitoring blood glucose as a non-enzymatic electrochemical sensor, exhibiting a sensitivity of 2.153 mA mM^−1^ cm^−2^ and a detection limit of 0.87 μM with a response time of 3 s. It measures glucose using the electrocatalytic properties of the composite, where Bi_2_O_3_ alternates between its oxidation states, and Ag promotes a parallel oxidation pathway, thereby facilitating the conversion of glucose to gluconolactone. This combined activity makes the sensor less likely to be affected by frequent interferents. It was tested on human serum samples, which yielded recovery results ranging from 90% to 95% [34]. This allows for high sensitivity and rapid detection of human serum. However, there are some obstacles to implementing it, as it works in a harsh alkaline solution instead of a typical physiological environment, and needs long-term studies and biocompatibility reassurance [34].

### 4.5. Optical Nanobiosensors

A 2014 study focused on developing a microgel-based fluorescent nanobiosensor for continuous glucose monitoring under normal physiological conditions. The researchers have incorporated G1.0 Polyamido amine (PAMAM) dendrimers as an intrinsic, anti-photobleaching fluorescent probe embedded within a poly(*N*-isopropylacrylamide-DMAEMA-AAPBA) microgel network, which swells upon binding to glucose. When glucose levels reach clinically significant levels (0–20 mM), the microgel expands, increasing the emission of blue fluorescence at 425 nm, which facilitates real-time monitoring of blood glucose. The PAMAM fluorophore provides greater photostability to other optical sensors and lower toxicity rates compared to QD systems. Moreover, the phenylboronic acid helps increase specificity and decrease interaction with other unwanted molecules in the blood. Most importantly, the microgel can maintain its structure in vivo and generate fluorescence, which can be detected transdermally, allowing for a minimally invasive glucose monitor with continuous monitoring capabilities [35].

The advancement of carbon-based nanomaterials, such as graphene, carbon nanotubes (CNTs), and carbon quantum dots (CQDs), has undergone significant evolution over time, and these systems now exhibit high sensitivity and reliability compared to other nanosystems. Graphene-based electrodes combined with catalytic co-materials have yielded exceptionally high electrochemical sensitivities. A study that developed a sensor using graphene-fibre/Au/Ni(OH)_2_ composites showed a sensitivity of around 1095.6 µA·mM^−1^·cm^−2^ over a 5 µM–2.2 mM glucose range, which is suitable for physiological glucose detection levels [36]. Other promising results are with the use of fluorescence-based carbon-dot sensors using CQDs embedded in molecularly imprinted polymer matrices, which have shown a limit of detection (LOD) of 29.4 nM in intracellular fluid mimicking solution with linear detection from 25 nM to 25 mM and a strong correlation (R^2^ = 0.9884), which shows the high sensitivity and very wide range of concentration detections with this combination detector system, which can help in implementing optical glucose sensing in the physiological systems as it can cover wide range of concentration [37]. This can be attributed to the properties of carbon dots, which have high photostability, tunable fluorescence emission, water dispersibility, and, additionally, low toxicity, making them suitable for in vivo studies [38]. Looking at the big image for carbon-based nanomaterials, specifically graphene composites, CNTs, and CQDs, we see their potential in delivering ultra-low detection limits, high sensitivity, and robust performance results when it comes to both electrochemical and fluorescence-based glucose sensing methods

## 5. Enzymatic Detection of Glucose

The enzymatic detection of glucose can be performed with the measurement of a few enzymes. These enzymes include GOx, Glucose Dehydrogenase (GDH), and Hexokinase.

### 5.1. Glucose Oxidase Method

Glucose Oxidase is used alongside peroxidase to yield glucose measurement. GOx is an enzyme that catalyzes the oxidation of beta-D-Glucose into gluconic acid and the conversion of oxygen into hydrogen peroxide (H_2_O_2_). Peroxidase (POD) is used to convert H_2_O_2_ into water and oxygen. The oxygen then reacts with 4-aminoantipyrine and phenol to form quinoneimine which has a distinct colour. The intensity of the colour is then used to measure glucose concentration [39]. The advantage of the GOx-POD method is that it is a cheap and simple test to conduct with high specificity for glucose. However, there are many interfering factors such as vitamin C, bilirubin, and uric acid which may limit its use [40].

The GOx-POD method has been used specifically in optical assays; however, the application of GOx in glucose monitoring has been extensively used in electrochemical monitoring. GOx-based electrochemical sensors are categorized into 3 generations based on the method of electron transfer between the enzyme and electrode. The first-generation GOx-based glucose sensors monitored the amount of O_2_ consumed or the amount of H_2_O_2_ produced. They contained electrodes which reacted with O_2_ or H_2_O_2_ to calculate the amount of glucose in a sample [41]. These sensors were first proposed in theory in 1962 and eventually commercialized in 1972. This generation of GOx-based sensors suffered from interference from a variety of substances, and required a high operation potential when oxidizing hydrogen peroxide [42]. It should be noted that this high potential applies specifically to hydrogen peroxide oxidation, whereas oxygen reduction in first-generation sensors occurs at relatively low cathodic potentials (approximately −0.2 to −0.3 V vs. Ag/AgCl) [43,44]. Second-generation GOx-based glucose sensors have redox mediators between enzymes and electrodes and are aided with the electron exchange process. As a result of these mediators, as glucose is introduced in a sample, the amount of current in the electrodes would increase [41]. Second-generation GOx-based sensors were demonstrated in the 1970s; however, in the 1980s, a mediator-based second-generation sensor was commercialized, marking a milestone in diabetes care [42]. Third generation GOx-based glucose sensors use engineered enzymes for direct electron transfer with electrodes. The enzymes use flavin adenine dinucleotide (FAD) as a subunit, and the nanoelectrodes are coupled with GOx which allows FAD to directly exchange electrons [41]. Third-generation GOx-based sensors have been experimented in implantable devices for continuous glucose monitoring, though no third-generation GOx-based implantable device exists on the market today [42].

### 5.2. Glucose Dehydrogenase Method

GDH is an important enzyme used in alternative glucose sensors as it is responsible for the oxidation reaction of glucose into gluconolactone alongside an electron carrier. Importantly, these sensors are not susceptible to interference from oxygen, unlike the GOx-based sensors. There are three types of GDH sensors: PQQ-dependent-GDH, NAD-dependent-GDH, and FAD-dependent-GDH. All 3 have similar mechanisms in which they use their cofactors (PQQ, NAD, FAD) to undergo redox reactions and transfer electrons from glucose. For example, NAD-dependent-GDH catalyzes the oxidation of beta-D-glucose into gluconolactone and the conversion of NAD+ into NADH, and the amount of NADH made is proportional to the amount of glucose [40]. PQQ-dependent-GDH can use different electron acceptors as co-substrates such as *N*-methylphenazonium (NMP) [45]. Disposable GDH-based sensors were introduced as an alternative to disposable GOx-based sensors, while modern CGM devices continue to use GOx. GDH-based sensors are currently used in point-of-care testing in both inpatient and outpatient settings [42].

The main advantage of the GDH method is that it is specific for glucose and its independence from oxygen which helps ensure results are accurate. FAD-GDH sensors are the newest form of GDH-based sensors and are found to be more specific than the others but have not been tested in the long-term [46]. GDH-based sensors offer quick reliable information at home for patients and only require a small blood sample [47]. There are a few disadvantages of GDH-based glucose sensors. For example, PQQ-GDH sensors have decreased selectivity and stability, and NAD-GDH sensors require cofactors that undergo irreversible redox reactions [40]. The FAD-GDH-based system has less interference from other sugars like maltose but still shows interference from sugars such as D-xylose [48].

### 5.3. Hexokinase Method

Hexokinase is an enzyme involved in the glycolytic pathway. It is responsible for the phosphorylation of glucose into glucose-6-phosphate (G6P) with the use of ATP. The G6P is generated then undergoes a reaction with NAD+ through glucose-6-phosphate dehydrogenase (G6PD) to produce NADH. The produced NADH is then used to calculate the concentration of glucose in a sample [49]. The main advantage of hexokinase is that it is very specific to glucose and very accurate and is usually used as the reference model for plasma glucose concentration [49]. The main disadvantage of hexokinase is that it takes a long time due to the deproteinization process, and it can be susceptible to some interference [49]. The merits and demerits of the various enzymatic glucose detection methods are summarized in Table 1. 

It is crucial to distinguish between electrochemical interferents and enzyme-substrate specificity. Electrochemical interferents specifically refer to redox-active species present in the biological fluids. For instance, ascorbic acid, uric acid, and acetaminophen can be oxidized or reduced at the electrode surface and generate spurious currents [42,50]. Whilst enzyme-substrate specificity relates to the intrinsic biochemical selectivity of the recognition enzyme toward glucose. GOx is generally considered highly specific for β-D-glucose, whereas certain GDH variants may exhibit cross-reactivity with other sugars such as maltose or xylose [51]. These two forms of interference arise from distinct mechanisms and should therefore be evaluated separately when comparing glucose sensing technologies.

## 6. Non-Enzymatic Detection Methods

### 6.1. Non-Enzymatic Detection

Aside from enzymatic detection, non-enzymatic-based sensors have shown promise. Recent research has shown a strong interest in these methods, particularly those that use nanomaterials designed to detect specific analytes. Non-enzymatic biosensors work by directly oxidizing glucose on an electrode with electrocatalytic features and a transition metal center. Developing non-enzymatic electrodes that are both efficient and effective is essential to identify various substances in organisms. When compared with enzymatic glucose sensors, non-enzymatic glucose sensors are more affordable and have a higher lifetime because they do not need expensive enzyme purification, immobilization, or cold-chain storage. They are also less affected by denaturation and oxygen dependence, which makes them more stable and increases the linear detection range [52].

Non-enzymatic methods, which involve direct glucose oxidation on electrodes, are attracting more attention. These non-enzymatic glucose monitoring methods are fast, sensitive, cost-effective, and reliable [53]. Enzymatic sensors are less stable and more expensive due to their reliance on biological components, while non-enzymatic glucose sensors are more stable and reliable as they are independent of enzymes. Given their several advantages, non-enzymatic detection methods are becoming more important in glucose monitoring for diabetes control [54]. However, despite strong laboratory performance, most non-enzymatic glucose sensors are still in the prototype or pre-clinical stage. Key barriers to translation into clinical practice include less selectivity in complex biofluids, being more likely to be affected by other substances (like ascorbate/uric acid), electrode surface fouling and signal drift over time. Another challenge is limited performance at physiological (near-neutral) pH compared to alkaline test conditions. There are also problems with reproducible large-scale fabrication and standardization [55]. Certain nanomaterials, such as platforms or catalysts, can improve the sensitivity and specificity of these sensors. These methods improve detection precision, sensitivity, and consistency using diverse principles and components. Key concepts, components, and methodologies in well-known non-enzymatic detection methods are discussed in the following section [56].

### 6.2. Electrochemical Methods in Non-Enzymatic Glucose Detection

Electrochemical methods stand out among non-enzymatic methods of glucose detection due to their simplicity and effectiveness. Electrochemical glucose sensors are based on the direct oxidation of glucose on electrode surfaces; this generates an electrical signal indicating the changes caused by glucose [55]. Electrochemical measurement offers a fast response time, great sensitivity and selectivity, and is not reliant on big analytical instruments. Furthermore, it is useful for real-time and continuous monitoring of any compound of interest. Given its many advantages, electrochemical methods are frequently used in a variety of industries including culinary arts, biomedical applications, and environmental detection [57].

The three-electrode system is commonly used in practical electrical analysis and detection [18]. Often, elements like metal, metal oxide, or carbon make up the working electrode. In electrochemical measurements, the use of a bare electrode can restrict its application since direct contact between reactant molecules, and the electrode surface limits its application. Functional nanomaterials are often used to modify electrodes to increase selectivity towards glucose. Electrochemical measuring techniques are used to generate signals after detecting the molecules on the electrode surface. These signals include important details on the target molecule, including the concentration. Metal nanoparticles, graphene, and carbon nanotubes are common materials utilized to make electrochemical glucose sensors [42]. Mechanisms underlying the interaction between glucose and these materials allow the generation of an electrochemical signal that precisely matches glucose concentration. The catalytic properties of the electrode materials allow the oxidation of glucose on their surface. Usually, the process of electrochemical glucose oxidation consists of the following phases. First, adsorption lets glucose molecules adhere to the surface of the electrode. Second, the glucose molecules oxidize and move electrons to the electrode. Finally, the electron transfer mechanism generates an electric current, and the signal generated is proportional to the glucose concentration [18].

### 6.3. Metal-Based Electrodes in Non-Enzymatic Glucose Detection

Platinum (Pt) has shown to be used as a metal-based glucose detector. Pt is a noble metal with excellent catalytic activity in the oxidation of glucose. The reaction process begins with the absorption of glucose onto the surface of Pt, then in dehydrogenation (glucose oxidation) and consequent electron transfer. A key limitation is that chloride ions can adsorb on Pt surfaces and poison catalytic sites. To fix this, nanostructuring and electrode design methods are commonly used to make the catalysts more stable and less likely to be poisoned [51]. In comparison, first-generation GOx sensors that measure enzymatically generated H_2_O_2_ typically operate at +0.6 V versus Ag/AgCl, whereas mediator-wired GOx systems are capable of reducing the applied potential by about −0.2 to +0.2 V versus Ag/AgCl (depending on the mediator) [50,58,59]. Conversely, many non-enzymatic electrochemical glucose sensors work in alkaline electrolytes at relatively positive (anodic) working potentials. For example, Au/Ni/Pt-type catalysts work at roughly +0.4 to +0.6 V against Ag/AgCl (e.g., Au nanorods on Ni foam work at 0.6 V vs. Ag/AgCl) [60]. When working at greater anodic potentials, the chance of unwanted side electrochemical oxidation from interfering species goes up compared to enzymatic or low-potential mediated systems [51]. Gold-based electrode that can be used as a glucose sensor as it has great catalytic activity. The reaction involves the attachment of glucose to the gold surface and its subsequent conversion to glucose oxide, which is aided by the enormous surface area of nanostructured gold. Using different metals to create alloys of Pt can greatly increase the durability and efficiency of catalysts taking advantage of strong synergistic effects. The surface of the alloy is made to create many active sites, which enhance the absorption and oxidation of glucose [55].

Carbon-based electrodes are another type of electrode used in non-enzymatic glucose detection, a common example being graphene. Graphene is highly flexible with great surface area, good electrical conductivity, and great chemical stability. The reaction consists of the binding of glucose to graphene’s surface then undergoing oxidation. The sensitivity of the sensor is enhanced by graphene’s high electron transfer rate [61]. Carbon nanotubes (CNTs) are yet another carbon-based electrode used in glucose sensing. CNTs have excellent electron transport properties and can be modified to improve glucose detection. Glucose is adsorbed onto the CNT’s surface and then oxidized. Better electron transport made possible by CNT configuration results in higher sensor performance [53].

**Figure 1:**
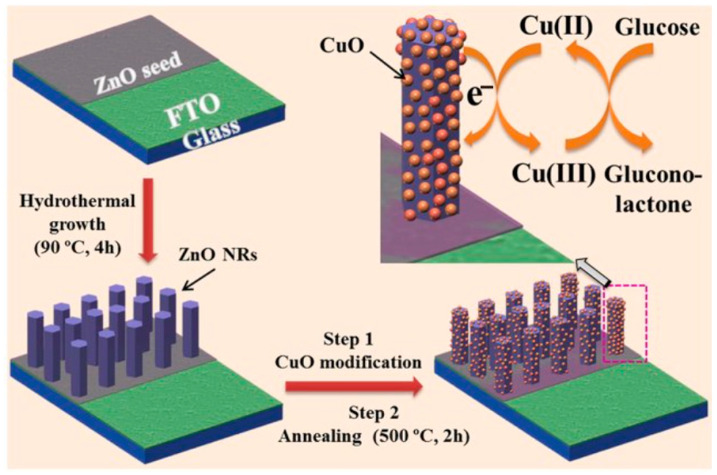
Schematic illustration of a non-enzymatic glucose sensor electrode fabrication and its application in glucose detection. Adapted from Ahmad et al., Scientific Reports, 2017, under CC BY 4.0 [63].

Metal oxides and hydroxides are another alternative non-enzymatic glucose detection method, an example being nickel oxide, as illustrated in Figure 1. Given that nickel and its derivatives have great catalytic efficiency in the oxidation of glucose, their use has been quite broad. Nickel hydroxide (Ni(OH)_2_) is electrochemically oxidised to nickel oxyhydroxide (NiOOH) in an alkaline solution. NiOOH is the active ingredient in the catalyst. NiOOH then changes glucose into gluconolactone and goes back to Ni(OH)_2_. The electron transfer that happens during this process creates a current that may be measured. Copper and its compounds (CuO and Cu(OH)_2_) also affect glucose oxidation by acting as catalysts. Cu(II) changes to Cu(III) by a sequence of chemical reactions. This process oxidises glucose to gluconolactone and creates an electric current [62]. Metal–organic frameworks (MOFs) are another component utilized in non-enzymatic glucose sensing. MOFs have a large surface area and can be designed to have specific pore structures, making them ideal for glucose sensing. Glucose is absorbed by the MOF surface and interacts with the metal centers during the reaction, resulting in oxidation. Absorption and catalytic effectiveness are much improved by the large surface area and porous structure [56].

### 6.4. Optical Methods

Optical glucose sensors monitor glucose levels through the interaction of light and glucose molecules. These sensors enable direct and real-time detection, making them ideal for speedy and enzyme-free measurements. They detect changes in optical properties, such as fluorescence intensity, surface plasmon resonance (SPR), or Raman scattering, that correspond to glucose levels in the sample [64]. Fluorescence-based glucose sensors measure glucose levels by either FRET or competitive binding processes. The photoluminescence (PL) signal in ZnO-based fluorescent glucose sensors may change in different ways depending on how the sensor is made. In enzyme-linked forms, glucose oxidase produces H_2_O_2_ during the oxidation of glucose. This H_2_O_2_ can then stop ZnO photoluminescence (collisional quenching), which makes it possible to measure glucose levels by looking at variations in PL intensity [65]. Metal nanoparticle-containing materials, such as ZnO nanorods or nanogels, are commonly used in these sensors. By increasing the electrostatic interactions with glucose molecules, ZnO nanorods’ high isoelectric point (IEP) helps them to absorb glucose. Variations in PL spectra due to glucose adsorption can be used to monitor glucose levels [66].

Fluorescent glucose sensors with high sensitivity and low limit of detection (LOD) can be created utilizing ZnO nanotubes (NTs) manufactured on low-cost printed circuit boards (PCBs) [29]. Real human blood samples were used in an experimental evaluation on a glucose sensor built of ZnO nanorods. The sensor’s performance was evaluated by comparing PL quenching glucose concentrations to hospital clinical data. The results showed a significant correlation (correlation factor of 0.99) between the PL quenching data and the clinical measures, therefore suggesting that the sensor might be applied for clinical monitoring of glucose concentrations in human blood. The usefulness and accuracy of using ZnO nanorod-based sensors for rapid glucose detection in clinical environments are highlighted in this study [67].

SPR sensors detect changes in refractive index near the sensor surface caused by the adherence of glucose molecules to a surface modified with certain functional groups. This approach monitors glucose levels in real time with great sensitivity. SPR sensor effectiveness is enhanced by combining Molybdenum diselenide (MoSe_2_) nanosheets with ZnO nanoparticles. The attachment of glucose molecules to a modified surface affects the local refractive index, therefore changing the SPR signal. Due to their large surface area and improved electron mobility, ZnO nanoparticles combined with MoSe_2_ nanosheets form a composite film that significantly increases the SPR signal. MoSe_2_ improves sensitivity and selectivity in glucose detection by increasing the signal’s specific surface area and electron mobility. Its measuring sensitivity is 72.17 nm/(mg/mL) and its detection limit is 4.16 (μg/mL) [68].

Raman spectroscopy is a technique used to measure the vibrational patterns of glucose molecules. Surface-enhanced Raman Scattering (SERS) methods magnify the Raman signal, therefore allowing a high degree of specificity to detect low levels of glucose. This method is useful since it is more specific and can provide detailed molecular information. Metallic nanoparticles like gold (Au) and silver (Ag) boost Raman signals due to their local surface plasmon resonance (LSPR), which facilitates glucose molecule interaction. Raman sensors use glucose’s unique vibrational patterns. The strength of the Raman signal grows proportionally with the concentration of glucose, making detection highly sensitive. The resonance of metal nanoparticles enhances free electron mobility, enhancing Raman signals from analyte molecules [56].

### 6.5. Thermal Methods

Thermal glucose sensors identify variations in heat generated by glucose oxidation. These sensors take advantage of the exothermic nature of the glucose oxidation reaction. The oxidation of glucose releases heat exactly proportional to the concentration of glucose. The thermal change is then monitored and linked to the glucose levels in the sample [61]. Non-enzymatic thermal biosensors use metal oxides or nanomaterials that react exothermically with glucose. For example, particular metal oxides can act as catalysts for the direct oxidation of glucose, resulting in the generation of measurable heat. The reaction consists of metal catalysts and the thermal transducer of the sensor that detects the generated heat, which allows an indirect measurement of glucose levels [69].

### 6.6. Magnetic Methods

Magnetic glucose sensors detect glucose-induced magnetic changes. These sensors primarily use magnetic nanomaterials, such as nickel nanoparticles (NiNPs), which interact with glucose molecules. The interaction results in a detectable change in the material’s magnetic properties, which is proportional to the glucose concentration [70]. In alkaline media, nanocomposite NiNPs can be electrochemically converted to nickel oxyhydroxide (NiOH)_2_. This conversion increases the substance’s catalytic efficacy during glucose oxidation. Significant electrocatalytic activity of the (NiOH)_2_ allows the oxidation of glucose molecules on the electrode surface. This reaction generates a measurable electrical signal that is proportional to the concentration of glucose. NiNPs’ magnetic properties help the sensor to be stabilized. When the NiNPs/3D-PMG nanocomposite is coupled with a magnetic electrode, it may be easily immobilized and removed from the electrode surface by using an external magnetic field. This feature helps the sensor become reusable and stable [71].

NiNPs combined with the three-dimensional porous graphene-like material have a synergistic impact that enhances the sensor’s overall efficacy. The graphene-like structure has a large surface area and high conductivity. The (NiOH)_2_ substance has catalytic properties. These combined features contribute to the glucose detection system’s high sensitivity, quick response time, and the ability interference resistance [62]. Non-enzymatic glucose sensing is still developing constantly. To further increase the sensitivity, selectivity, and stability of non-enzymatic glucose sensors, it is possible that one of the upcoming advances will involve the production of unique nanomaterials and hybrid structures.

## 7. Glucose Detection by Breath and Saliva

### 7.1. Overview of Non-Invasive Methods of Glucose Detection

Non-invasive glucose detection methods represent an emerging technology for more convenient and inexpensive point-of-care testing for glucose levels. These methods depend on the fact that diabetic patients’ health status can be detected and monitored by analyzing biomarkers present in their body fluids such as saliva, tears and breath. For decades blood samples have been used to monitor glucose levels; however, concerns over patient discomfort and pain have led to a shift towards non-invasive methods [72].

### 7.2. Saliva-Based Biosensors

Saliva is an important body fluid that has been utilized in toxicology and forensic medicine as it correlates with different conditions. One of these conditions is diabetes, as evidence demonstrates a significant correlation between increased fasting blood glucose (FBG) levels and fasting salivary glucose (FSG) levels [65]. Although normal glucose content in saliva for non-diabetic patients is much lower than in blood (0.5–1.00 mg/dL compared to 70 to 99 mg/dL), recent techniques have been developed to enhance the detection of salivary glucose content by utilizing both enzymatic and non-enzymatic biosensors [73].

**Figure 2:**
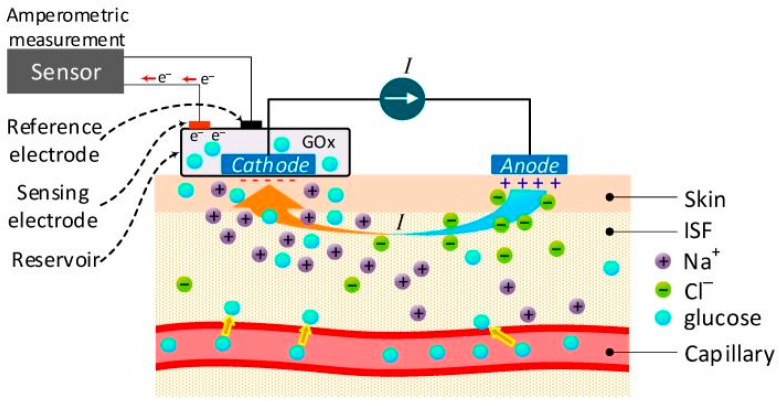
An amperometric glucose sensor using GOx to detect interstitial glucose. Adapted from Sensors 2019;19(4):800, licensed under CCBY4.0 [74].

Enzymatic biosensors aim to enhance the detection of small amounts of glucose present in saliva, and they have the best selectivity and sensitivity for glucose compared to other biosensors. Organic Electrochemical Transistors (OECTs) are considered a promising method of monitoring glucose levels within tears. The function of OECTs depends on the enzyme glucose oxidase (GOx) to oxidize glucose and subsequently measure the hydrogen peroxide (H_2_O_2_) produced during the reaction, as illustrated in Figure 2 [72]. However, evidence has demonstrated that enzymatic biosensors are highly susceptible to changes in temperature, pH, and humidity [72]. Enzymatic biosensors are susceptible to instability which has led to the consideration of non-enzymatic sensors. Non-enzymatic sensors use metals such as silver, gold, and copper as catalysts. These biosensors represent a promising field for investigation, as current sensors, despite their low sensitivity and selectivity, were able to distinguish between diabetic and non-diabetic individuals successfully. Sensors with copper oxides demonstrate even higher sensitivity because of their enhanced electrocatalytic activity to glucose oxidation [72].

Overall, saliva-based biosensors show potential as a non-invasive method for glucose detection, the major benefits being the convenience of saliva collection compared with invasive techniques that are used for blood collection and the relatively low cost of these biosensors; however the limited content in glucose levels in the saliva represents the major challenge as it limits their sensitivity [65].

### 7.3. Tears-Based Biosensors

Tear fluid has an important role as a protective film for the eye and it is produced by the lacrimal glands, and they are unique as the biomarkers in blood diffuse directly to them. Tears have attracted immense attention for glucose detection owing to being non-invasive and their strong correlation with blood glucose levels [75]. The glucose concentration in tear samples from non-diabetic healthy individuals is 3.6 mg/dL, whereas in diabetic patients, it is about 16.6 mg/dL [76].

Many types of tear-based biosensors were investigated utilizing both enzymatic and non-enzymatic techniques. The development was not only in enhancing the performance of the biosensors, but also in developing more convenient methods for tears collection and wearable devices. For example, contact lenses were developed that can respond to glucose concentration changes by creating osmotic pressure that is transmitted as shrinking or swelling of the lens which can be simply detected by mobile phone software [76]. Tears represent an excellent choice for glucose monitoring as they closely correlate with blood glucose levels; however, there are difficulties collecting tears given the small sample size and ease of evaporation. The development of tear-based biosensor wearable devices may resolve these difficulties [77].

### 7.4. Breath-Based Biosensors

The composition of exhaled breath can demonstrate the metabolic changes of the body and can be used for monitoring glucose levels. The principle is to observe the organic volatile components (VOC) in exhaled breath such as acetone. Acetone levels in exhaled breath are directly correlated with blood glucose levels. For a non-diabetic healthy individual, a normal level is 0.8 parts per million volumes (ppmv) whereas in a diabetic patient it is greater than 1.8 ppmv [78]. Various breath analysis methods were developed to monitor acetone levels taking advantage of mass spectrometers such as gas chromatography–mass spectrometry which has high sensitivity. However, these types of breath sensors are time-consuming, expensive and require trained operators [79]. These obstacles led to the development of portable diagnostic devices that provide real-time monitoring for acetone levels [80]. Overall, breath-based biosensors show great potential for glucose monitoring as with more development in portable devices with semiconductive metal oxidase as they can provide real-time monitoring at low cost. However, one main concern remains in obtaining a linear correlation between acetone levels and blood glucose levels under different experimental conditions [81].

### 7.5. Transformative Potential of Non-Invasive Biofluids

Non-invasive biofluids such as saliva, tears, sweat and urine have the potential to change glucose monitoring by removing the need for finger-prick sampling. These fluids can be obtained easily and comfortably and may produce improved adherence to regular monitoring. Advances in microfluidic structures and nanomaterial-based detection have increased the sensitivity of biosensors that operate at very low glucose concentrations, particularly in saliva and sweat where basal levels are significantly lower than in plasma [73]. Tear-based sensors demonstrate a strong correlation with blood glucose levels. They can be integrated into smart contact lenses or ocular patches, which allows continuous collection of tear film without altering the patient’s daily routine [76]. Sweat-based systems allow on-body tracking through wearable patches and may provide real-time glucose estimates during exercise or daily activity [82]. As these platforms continue to improve, non-invasive biofluids can reduce monitoring burden and provide more accessible, comfortable and sustainable glucose tracking for diabetic patients.

## 8. Challenges in Clinical Practice

### 8.1. Challenges in Clinical Practice with Glucose Detection Methods

Alongside the innovation in tech-based blood glucose detection exists a set of barriers to their application in clinical practice, including increases in cost, limited accessibility, inaccuracy, regulatory hurdles, and uncertainties around patient compliance. Understanding and addressing these challenges is critical for improving diabetes management moving forward and must be a point of emphasis for future developers and regulatory bodies to ensure seamless integration of these devices into the patient population.

The cost associated with glucose detection methods poses a significant barrier for many patients. The initial investment in advanced glucose monitoring systems requires substantial upfront expenses, as well as recurring costs for consumables such as test strips and sensors [83,84]. Furthermore, insurance and reimbursement issues also add to the financial burden as coverage varies widely, with high deductibles, copayments and coverage limits, leading to substantial out-of-pocket expenses [83,85]. This means that for uninsured patients of lower socioeconomic backgrounds, inconsistent monitoring and poorer health outcomes are almost inevitable. Accessibility issues are commonly reported in underserved populations residing in rural or remote areas with limited access to medical supplies and pharmacies [85]. These conditions force patients to set aside a significant amount of time and travel long distances to obtain glucose monitoring devices. However, these commitments are often viewed as unsustainable and costly, leading to infrequent monitoring and suboptimal diabetes management [85]. Technological barriers also play a role in limiting accessibility as modern CGM systems often rely on smartphone apps for data tracking and analysis, which not all patients can access due to a lack of smartphones, internet or technological literacy [86].

Inaccurate measurements also pose a significant barrier to the effective use of glucose monitoring devices, an issue attributable to several causes. Firstly, improper calibration or a lack of regular maintenance can lead to inaccurate blood glucose readings [22]. Secondly, user error, including incorrect application of the test strips or inadequate blood sampling can also result in inaccurate readings, particularly among elderly patients or those with limited dexterity [87]. Furthermore, environmental factors including temperature, humidity and altitude can affect the performance of glucose sensors, while physiological variations such as skin thickness, hydration levels, and circulation can impact the accuracy of CGM systems [87,88]. Although some human error is inevitable, most inaccuracies can be avoided with proper patient education around the factors mentioned.

### 8.2. Patient Compliance

Patient compliance refers to the degree of adherence to the given medical advice and treatment plans [89]. It is a vital factor that must always be considered in the management of many health conditions, including diabetes. One of the main aims of diabetic management is to achieve good metabolic control and minimize the risk of serious complications. Patient compliance is crucial in this process, and neglecting it can lead to significant clinical challenges. Factors that affect compliance include the ease of use of treatments, patient education, discomfort, and cost. Without compliance, there would be little to no improvement in metabolic control.

A study conducted at the outpatient clinic of Zagazig University Hospital assessed the compliance of 80 patients with type 2 diabetes mellitus [90]. The study found that low levels of compliance were primarily due to patients’ income and their degree of knowledge of their condition. This issue was especially evident amongst patients from low socio-economic backgrounds, many of whom were unemployed or illiterate. Lower-income patients often struggle to afford health insurance and treatment, while less educated patients may not fully comprehend the severity of their condition or the importance of adhering to their treatment plan.

Another study illustrated that according to Chinese guidelines only 56.7% of patients had adequate compliance and that factors such as the duration of diabetes and the use of oral hypoglycemic agents led to a meaningful increase in compliance [91]. This is reasonable given that the patients who have diabetes for a longer time would have had more time to adapt to managing their condition and gained more knowledge about necessity of adhering to their treatment plan. Furthermore, oral treatment seemed to be another important factor that led to increased compliance; this is most likely due to the increased convenience of oral treatment alongside it being cheaper. These studies identify the main factors that influence patient compliance as income, education, duration of diabetes, and route of treatment. Understanding these factors enables physicians to develop strategies to address these aspects, to minimize complications related to patient compliance. One such strategy entails the emphasis of patient education regarding their condition and treatment plans with the addition of the possible consequences of lack of adherence to the treatment plan.

### 8.3. Barriers to Clinical Adoption of Non-Invasive Glucose Sensors

Several barriers continue to limit the clinical use of non-invasive glucose sensors. The most significant issue is the very low glucose concentration in saliva, tears and sweat, which makes accurate measurement difficult and increases susceptibility to interference from other biomolecules [73]. These biofluids also vary significantly with hydration, temperature, pH and environmental conditions, which reduces stability and complicates calibration [12]. Another challenge is the inconsistent correlation between glucose levels in alternative biofluids and blood glucose during exercise, stress or rapid metabolic changes. These physiological differences can delay glucose equilibration and reduce reliability for real-time monitoring [92]. Many prototype sensors also experience material degradation, signal drift and limited durability when exposed to moisture, enzymes or sweat for extended use [29]. Regulatory approval is another obstacle because most non-invasive platforms do not consistently meet the accuracy standards required for medical decision making, such as ISO 15197 [87]. Improvements in sensor sensitivity, environmental stability and calibration algorithms are needed before these technologies can be widely adopted in clinical practice.

## 9. Future Perspective and Emerging Directions in Glucose Detection

### 9.1. Future Perspectives in Glucose Detection

Upcoming advancements in glucose-detecting technology have the potential to greatly enhance diabetes management and treatment progression. New glucose sensor materials and technologies are being tested to improve sensitivity, selectivity, and durability [18]. Wearable glucose monitors are getting more accurate and less invasive, with improvements in sensor membranes, calibration algorithms, and signal processing leading to enhanced precision in real-time monitoring [11]. These technologies, like CGMs, are improving to deliver real time, precise glucose measurements without finger pricks or invasive procedures [11]. Recent improvements favour non-invasive monitoring devices that use enhanced sensor technology and algorithms to measure glucose levels in the skin or interstitial fluid [82]. The KnowU sensor, developed by Know Labs, uses radio frequency sensors that interact with the dielectric properties of glucose molecules [93]. This non-invasive procedure eliminates the need for subcutaneous methods and is more comfortable. CGM advances aim to enhance patient adherence and diabetes management by providing easier-to-use and more precise glucose monitoring options [10].

### 9.2. Glucose Detection with Wearable Biosensors

Glucose detection is being integrated into smartphones and digital health systems. This allows fast data collection and ongoing monitoring, therefore enhancing the management of diabetes [76]. These devices allow doctors to remotely track wearable sensor data for quick treatment, and mobile devices coupled with digital platforms encourage patient compliance by offering immediate feedback and personalised insights [86]. Leading non-invasive glucose monitoring are wearable sweat-based glucose sensors. Utilising smart patches, wearable gear collects and analyses sweat using microfluidic platforms to show glucose levels [94]. Continuous glucose tracking from these devices and digital health systems helps patients better control their diabetic status.

Another painless approach to monitor glucose levels is via wearable tear-based glucose sensors. Recent technological development has created contact lenses and eyeglass-mounted sensors with optical and electrochemical detection capabilities [73]. These wearable ocular devices demonstrate a strong correlation with blood glucose levels and can integrate into digital health systems for continuous tracking [75]. Wearable saliva-based glucose sensors are intended to monitor glucose levels quickly and painlessly, eliminating the need for invasive procedures. Wireless mouthguards and intraoral sensors can measure glucose in saliva with growing accuracy, supported by advances in enzymatic and non-enzymatic detection chemistries [41]. These sensors connect to digital platforms for real-time glucose measurement and convenient diabetes management. Wearable microneedle-based interstitial fluid (ISF) glucose sensors enable continuous glucose level monitoring by extracting and analysing glucose from the fluid surrounding cells [95]. With real-time glucose readings, these smart wearable devices can link with digital health systems for the quick management of diabetes and other metabolic diseases. Microneedle platforms are increasingly studied for their minimal pain, biocompatibility and stable long-term performance [96].

### 9.3. AI and Machine Learning: Enhancing Glucose Monitoring Systems

Analysis of data from wearable glucose sensors using machine learning techniques is significantly improving glucose monitoring. These algorithms can detect subtle glucose changes and predict hypoglycaemia and hyperglycaemia using supervised and unsupervised learning [72]. Machine learning enhances glucose monitoring and early diabetes care by spotting patterns in real-time data [97]. AI-powered devices can inform users or healthcare professionals as soon as glucose levels exceed critical limits, improving diabetes control [97]. Wearable microneedles combined with closed-loop AI systems can adjust insulin delivery in response to glucose fluctuations, supporting personalised metabolic management [98]. AI methodologies also support diet optimisation by linking glucose responses to nutritional intake, allowing targeted dietary recommendations [98]. This integration guides food decisions, improving lifestyle and diabetes control.

### 9.4. Integration of Wearable Biosensors and Artificial Intelligence

Wearable biosensors can be linked with artificial intelligence systems to create personalised diabetes management platforms. These devices collect continuous physiological data, including glucose levels, activity, sleep patterns, temperature, and heart rate. Machine learning models can analyse this information and detect patterns that predict fluctuations in glucose levels. This includes forecasting hypoglycaemic or hyperglycaemic episodes, identifying lifestyle triggers, and providing individualised behavioural or therapeutic recommendations based on real-time physiological feedback. Artificial intelligence systems can also automate data interpretation and improve clinical workflow by allowing healthcare professionals to review long-term glucose trends remotely [96]. With increasing integration into smartphones and cloud-based monitoring systems, wearable biosensors combined with artificial intelligence may allow highly adaptive, patient-centred metabolic management for both type 1 and type 2 diabetes [99].

## 10. Current and Potential Breakthroughs

### 10.1. Use of Other Body Fluids for Glucose Measurements

There has been lots of research focused on the use of tears, ISF, and salvia for the measurement of glucose. For tears, models of contact lens glucose sensors have been researched and developed. However, they are limited due to low power supply and potentially blocking the user’s field of vision [41]. For saliva, mouthguard-type glucose sensors have been researched and developed, but both the saliva and tear-based glucose sensors are impractical for use due to the uncomfortably and potential optical and dental effects [41]. ISF-based glucose sensors have been developed as ISF has glucose levels similar to plasma. These ISF-based monitors exist as either continuous monitors or intermittent monitors. ISF may be a good method of glucose sensing as it can be easily retrieved and is affected less by infections. However, problems with ISF arise as there are delays in ISF glucose levels compared to plasma glucose levels which can cause sensors to fail [92].

Nanotechnology-based glucose sensors require further work to address limitations in stability, biocompatibility and fabrication cost. Current nanostructured electrodes and quantum dot-based systems are highly sensitive, although they often show vulnerability to temperature, humidity and pH fluctuations which limits their use outside controlled laboratory settings [18]. Improving the long-term stability of these materials and creating uniform large-scale production processes will be essential for clinical feasibility. Future research should prioritise hybrid nanomaterials that combine metal oxides, carbon-based nanostructures and polymer coatings to enhance selectivity and minimise interference from physiologic metabolites. Additional work is required to optimise integration with wireless platforms and smartphone-based analytics, which will allow truly non-invasive systems to become practical in everyday healthcare. Increasing affordability through simplified synthesis methods and scalable manufacturing should also be a major focus, especially for deployment in low-resource settings [29].

### 10.2. Non-Enzymatic Glucose Sensors

New technologies are being researched and developed into invasive and non-invasive glucose sensors. In non-invasive glucose sensors, research is being conducted into optical transducers that use properties of light to detect glucose, and transdermal sensors which use ionophoresis or sonophoresis in the detection of glucose. In invasive glucose sensors, research is being done into subcutaneous needles and micro dialysis [100]. Optical transducers are heavily researched using many different techniques. These techniques include thermal infrared, spectroscopy, fluorescence, and polarimetry. These methods may be used to measure glucose levels in the eye [100].

Transdermal sensors use reverse ionophoresis which is when two electrodes with a low current are applied to the skin which causes ions to move across the skin and for neutral molecules like glucose to move between both electrodes. This method collects a very low concentration of glucose which is a slight advantage as oxygen is not a hindrance, but these sensors have had trouble in the past detecting hyperglycemia [100]. Another method used in transdermal sensors is sonophoresis which is where ultrasound is used to increase permeability and ISF collection [100]. There are many new up and coming technologies in relation to the detection of glucose, and there is no doubt that newer and better technologies will be developed in the treatment and care of diabetes.

## 11. Conclusions

The advancements in glucose biosensor technologies, including enzymatic, non-enzymatic, and non-invasive methods and their effect in improving diabetic management were discussed in this paper. Historical contributions, from early chemical tests to sophisticated continuous glucose monitoring systems, only underscore the fact that much progress has been made in this field. From simple and qualitative methods to more accurate, real-time monitoring technologies, the evolution brings forth the growing capability of managing diabetes more effectively.

The current glucose detection biosensors are based on one of two methods: enzymatic and non-enzymatic. Enzymatic biosensors display good accuracy and selectivity for glucose, but their stability is often troubled. In comparison, the non-enzymatic method comprises different principles and elements, which result in the increment of precision, sensitivity, and continuity of glucose detection, making it more promising. New technologies of glucose detection such as non-invasive sensors and wearable devices, promise to enhance patient comfort and ease of use. Innovations like wearable sweat-based, tear-based, and saliva-based sensors are pushing the boundaries of non-invasive monitoring, aiming to provide continuous and convenient glucose tracking. Additionally, advancements in artificial intelligence and machine learning are set to revolutionize data analysis, improving the accuracy and timeliness of glucose monitoring and enabling better disease management.

Despite these achievements, some key challenges remain in terms of cost, accessibility, accuracy, and patient compliance. Overcoming those barriers lies in research and developing the technologies for glucose sensing with better systems of education and support of the patients. Overall, the future of glucose detection is promising, with continued innovations expected to further enhance diabetes management. The integration of advanced biosensors, digital health systems, and AI-driven solutions will likely lead to more accessible, effective and patient-friendly approaches to monitoring and treating diabetes.

## Figures and Tables

**Table 1:** Summary of the merits and demerits of the various enzymatic glucose detection methods.

Factors	GOx	GDH	Hexokinase
Sensitivity	Good	High	Extremely high
Specificity	High glucose specificity	Potential cross-reactivity depending on enzyme type (e.g., maltose, and xylose)	Extremely high
User-Friendliness	Good	Good	Not user-friendly. Longer detection time
Interference	Ascorbic acid, uric acid, and acetaminophen	Minimal electrochemical interference; enzyme-dependent sugar cross-reactivity	Minimal interference.
O_2_ Dependence	Yes	No	No

## Data Availability

No new data were created or analyzed in this study.

## Abbreviations

The following abbreviations are used in this manuscript:

AA	Ascorbic Acid
AI	Artificial Intelligence
ARM	Augmented Reality/Machine (context dependent)
CC	Cross-Correlation/Carbon Composite (context dependent)
CGM	Continuous Glucose Monitoring
CNT	Carbon Nanotubes
FAD	Flavin Adenine Dinucleotide
FPG	Fasting Plasma Glucose
FRET	Förster Resonance Energy Transfer
G6P	Glucose-6-Phosphate
GDH	Glucose Dehydrogenase
H_2_O_2_	Hydrogen Peroxide
IDF	International Diabetes Federation
ISF	Interstitial Fluid
IUPAC	International Union of Pure and Applied Chemistry
ML	Machine Learning
NAD	Nicotinamide Adenine Dinucleotide
NADH	Reduced Nicotinamide Adenine Dinucleotide
O_2_	Oxygen
OGTT	Oral Glucose Tolerance Test
PL	Phospholipid
POD	Peroxidase
PQQ	Pyrroloquinoline Quinone
QD	Quantum Dot
SPR	Surface Plasmon Resonance
WHO	World Health Organization
